# Impact of Rehabilitation Dose on Nutritional Status at Discharge from a Convalescent Rehabilitation Ward in Malnourished Patients with Hip Fracture

**DOI:** 10.3390/healthcare9060722

**Published:** 2021-06-12

**Authors:** Yusuke Ito, Hidetaka Wakabayashi, Shinta Nishioka, Shin Nomura, Ryo Momosaki

**Affiliations:** 1 Department of Rehabilitation, Beppu Rehabilitation Center, Oita 874-8611, Japan; gairai-reha@brc.or.jp; 2 Department of Rehabilitation Medicine, Tokyo Women’s Medical University Hospital, Tokyo 162-0054, Japan; noventurenoglory@gmail.com; 3 Department of Clinical Nutrition and Food Service, Nagasaki Rehabilitation Hospital, Nagasaki 850-0854, Japan; shintacks@yahoo.co.jp; 4 Department of Rehabilitation Medicine, Mie University Graduate School of Medicine, Mie 514-8507, Japan; momosakiryo@gmail.com

**Keywords:** malnutrition, hip fracture, rehabilitation dose, older adults, convalescent rehabilitation ward

## Abstract

The object of this study is to determine the impact of the rehabilitation dose on the nutritional status at discharge from a convalescent rehabilitation ward in malnourished patients with hip fracture. This retrospective case-control study involved malnourished patients with hip fracture aged 65 years or older who had been admitted to a convalescent rehabilitation ward and whose data were registered in the Japan Rehabilitation Nutrition Database. The primary outcome was nutritional status at discharge. Patients were classified according to whether nutritional status was improved or not at discharge, according to the Mini Nutritional Assessment-Short Form^®^ (MNA-SF) score. The association between improved nutritional status and rehabilitation dose was assessed by a logistic regression analysis. Data were available for 145 patients (27 men, 118 women; mean age 85.1 ± 7.9 years). Daily rehabilitation dose was 109.5 (median 94.6–116.2) min and the MNA-SF score at admission was 5 (median 4–6). Nutritional status was improved in 97 patients and not improved in 48. Logistic regression analysis showed the following factors to be independently associated with nutritional status at discharge: Functional Independence Measure score (OR 1.042, 95% CI 1.016–1.068), energy intake (OR 1.002 CI 1.000–1.004), daily rehabilitation dose (OR 1.023, 95% CI 1.002–1.045), and length of hospital stay (OR 1.026, 95% CI 1.003–1.049). The daily rehabilitation dose in malnourished patients with hip fracture may positively impact nutritional status at discharge.

## 1. Introduction

The rapidly aging population in Japan has been accompanied by a steady increase in the number of hip fractures in the elderly [1,2]. These fractures are burdensome for both patients and the health care system, particularly in terms of the ongoing need for nursing care. One solution to this problem is intensive rehabilitation starting in the early postoperative period so that patients can learn to walk again and optimize their ability to perform activities of daily living (ADL). A greater rehabilitation dose has been shown to improve the Functional Independence Measure (FIM) score, increase the likelihood of return to home, and shorten hospital stays [3]. However, 14–65% of patients with hip fracture are malnourished [4,5], and aggressive rehabilitation without nutritional care may worsen nutritional status. Energy intake and macro- or micronutrient needs are increased during rehabilitation, which may increase risks in patients who may already have nutrient deficiencies [6,7,8,9]. Therefore, rehabilitation should be implemented with an understanding of both nutritional status and physical function.

The appropriate rehabilitation dose that takes nutritional status into account in patients with hip fracture is not known. Previous studies have reported that improved nutritional status in patients with hip fracture contributes to recovery of the ability to perform ADL at discharge and that an increased rehabilitation dose improves outcomes [3,10]. However, to our knowledge, there are no reports on the relationship between rehabilitation dose and nutritional status at discharge.

Therefore, the purpose of this study was to clarify the effect of rehabilitation dose during convalescent rehabilitation on nutritional status at discharge in malnourished patients with hip fracture.

## 2. Materials and Methods

### 2.1. Japan Rehabilitation Nutrition Database

This retrospective case-control study included patients with hip fracture whose data had been registered in the Japan Rehabilitation Nutrition Database (JRND). This database is managed by the Japanese Association of Rehabilitation Nutrition and was created to disseminate clinical research findings on rehabilitation nutrition worldwide. The database contains data on patients with pneumonia in acute care hospitals and those with stroke or hip fracture treated in convalescent rehabilitation wards. As of 31 March 2018, there were 11 participating centers and 1100 case registrations. Data recorded in the JRND at admission are age, sex, dates of onset and admission, diagnosis, type of surgery, Charlson Comorbidity Index (CCI) score [11], pre-fracture public long-term care insurance certification, medications, serum albumin level, FIM score, Food Intake LEVEL Scale (FILS) score [12], level of consciousness, height, body weight, Mini Nutritional Assessment-Short Form^®^ (MNA-SF), energy intake, dietary form, and route of feeding. Energy intake was evaluated as the mean over the first week after admission. The method for estimating energy intake was not specified when it was registered in the database. Most hospitals in Japan plan menus with commercially available software, incorporating the Standard Tables of Food Composition in Japan—2015 [13]. The amount of each food item consumed by the patient is visually observed by nursing staff or a registered dietitian and is recorded as a percentage. These data are then converted into energy intake by a registered dietitian using the standard tables [14]. Data recorded at discharge are the date of discharge, destination, total rehabilitation dose, FIM and MNA-SF scores, and weight. The study was approved by the Jikei University School of Medicine (approval number: 27-150 (8035)). Because only anonymized clinical data are registered in the JRND, informed consent was obtained via an opt-out procedure. All participating centers provide information about the JRND and the opt-out procedure to all patients. Patients can withdraw from the registry at any time.

### 2.2. Participants

This study involved 234 patients with hip fracture at nine centers who were admitted to a convalescent rehabilitation ward. Patients younger than 65 years, those with an MNA-SF score of ≥8 at admission, and those with missing data were excluded. Patients who had spent >60 days in an acute care hospital after hip fracture, and those with a convalescent rehabilitation stay of ≥91 days, were also excluded because they were not covered by public medical insurance for acute care or rehabilitation beyond these times.

### 2.3. Main Outcome Measurement

The primary outcome was nutritional status at discharge from a rehabilitation ward. The MNA-SF was used to assess nutritional status [15,16,17]. This instrument comprises six questions concerning food intake, weight loss, gait, mental stress or acute illness, neurological and psychological problems, and body mass index. It is a validated nutritional screening tool that can identify patients aged 65 years or older who are malnourished or at risk of malnutrition; a score ≥12 indicates good nutrition, 8–11 as at risk, and ≤7 as malnutrition. In this study, we classified nutritional status at discharge as improved (MNA-SF score ≥8) or not improved (MNA-SF score ≤7).

### 2.4. Rehabilitation Dose

In the convalescent rehabilitation ward, public medical insurance covers individual rehabilitation for patients with hip fracture provided by physical therapists, occupational therapists, and speech–language–hearing therapists for nine units per day (1 unit = 20 min), 7 days per week, for up to 90 days. The time allocation for each type of rehabilitation can be tailored according to the needs of the individual patient. In this study, the daily rehabilitation dose was defined as the total amount of time spent on physical therapy, occupational therapy, and speech–language–hearing therapy during hospitalization, divided by the length of the hospital stay.

### 2.5. Statistical Analysis

The items surveyed were age, sex, CCI score, FIM score, MNA-SF score, FILS score, energy intake, daily rehabilitation dose, length of hospital stay, and the number of hospital beds in the convalescent rehabilitation wards at admission. The unpaired *t*-test was used to examine continuous variables with a normal distribution, and the Mann–Whitney U test was used to examine continuous and ordinal variables that were not normally distributed. The chi-square test was used for nominal variables. Next, the effect of the rehabilitation dose on nutritional status at discharge was assessed by logistic regression analysis. For the dependent variable, the patients were assigned to two groups: one group with no change in MNA-SF status at discharge and another group with improved MNA-SF status at discharge. To account for multicollinearity, we checked whether each variable had a Spearman’s rank correlation coefficient with an absolute value of <0.7. All statistical analyses were performed using IBM SPSS Statistics version 18 (IBM Corp., Armonk, NY, USA). Statistical significance was set at 5%.

## 3. Results

**Figure 1:**
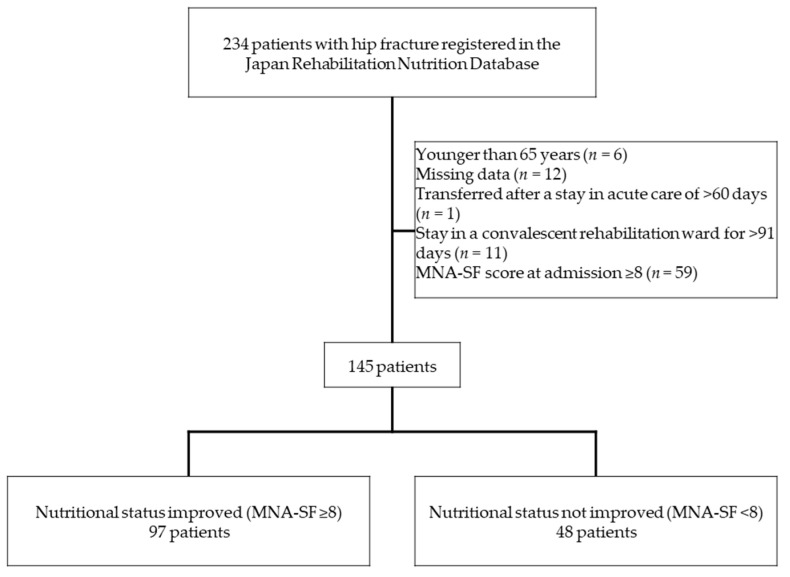
Study design. MNA-SF, Mini Nutritional Assessment-Short Form.

Data for a total of 234 patients with hip fracture at nine centers were registered in the JRND. After exclusion of patients younger than 65 years (*n* = 6), those with missing data (*n* = 12), those who were transferred for rehabilitation after spending >60 days in an acute care ward (*n* = 1), those whose stay in a convalescent rehabilitation ward was >91 days (*n* = 11), and those with an MNA-SF score of ≥8 at admission (*n* = 59), data of 145 patients at nine centers were ultimately available for analysis (Figure 1).

The 145 patients (27 men, 118 women; mean age 85.13 ± 7.94 years) received a daily rehabilitation dose of 109.49 (median 94.61–116.21) min and had an MNA-SF score of 5 (median 4–6) at admission. There were 97 patients in the improved group and 48 in the not improved group. In the improved group, 88 patients (91%) were deemed to be at risk of malnutrition (MNA-SF score 8–11) and 9 (9%) were considered to have good nutritional status at discharge (MNA-SF score ≥12).

The results for each survey item and the results of univariate analysis for the two groups are presented in Table 1. The group with improved nutritional status had a younger mean age (84.2 years vs. 86.9 years, *p* = 0.009), higher median FIM score on admission (71 vs. 46, *p* < 0.001), higher MNA-SF score on admission (median 5 vs. 4, *p* < 0.001), and higher FILS score on admission (9 vs. 8, *p* < 0.001). This group also had a higher median energy intake (1400 vs. 1200 kcal/day, *p* < 0.001), higher median daily rehabilitation dose (110.6 vs. 101.11, *p* = 0.008), and higher median number of hospital beds (206 vs. 60, *p* = 0.012). However, there was no difference in CCI (*p* = 0.225) or length of hospital stay (*p* = 0.696). All variables had a Spearman’s rank correlation coefficient with an absolute value less than 0.7 (Table 2), indicating that there was no multicollinearity in the logistic regression analysis.

The results of the logistic regression analysis are shown in Table 3. Among the covariates selected by the forced entry method, the FIM score on admission (odds ratio [OR] 1.042, 95% confidence interval [CI] 1.016–1.068), energy intake (OR 1.002, CI 1.000–1.004), daily rehabilitation dose (OR 1.023, 95% CI 1.002–1.045), and length of hospital stay (OR 1.026, 95% CI 1.003–1.049) were extracted as independent predictors of improvement in nutritional status. The odds ratio of a 20 min (1 unit) increase in daily rehabilitation dose for clinical improvement in nutritional status was 1.583 (95% Cl 1.043–2.401, *p* = 0.031).

## 4. Discussion

In this study, there were two notable clinical findings regarding nutritional status and appropriate rehabilitation dose. First, in malnourished patients with hip fractures admitted to the convalescent rehabilitation ward, an increased daily rehabilitation dose was associated with improved nutritional status at discharge. Second, the nutritional status of 67% of malnourished patients with hip fracture admitted to the convalescent rehabilitation ward improved while in the hospital.

Convalescent rehabilitation wards in Japan aim to prevent patients from becoming permanently bedridden and to allow them to return to home by improving their independence in ADL. Physical therapists, occupational therapists, and speech–language–hearing therapists provide up to 180 min per day of intensive rehabilitation from the first day of hospitalization onward. However, it has been reported that 26.2% of patients with hip fracture admitted to a convalescent rehabilitation ward have insufficient energy intake [18]. If energy intake is less than energy expenditure, aggressive exercise therapy is unlikely to be useful and may actually worsen nutritional status. In this study, nutritional status was thought to have improved by increasing the rehabilitation dose after ensuring the level of nutrition necessary for rehabilitation. Even if aggressive nutritional therapy was provided for patients with malnutrition, if exercise therapy was not implemented at the same time, there would be an increase in body fat mass, which would inhibit performance of ADL. Therefore, the effectiveness of nutritional therapy depends on the amount of exercise therapy administered. Previous studies have reported that exercise therapy in conjunction with nutritional therapy leads to greater synthesis of muscle proteins [19,20] and improved nutritional status [21,22]. It has also been shown that an increased rehabilitation dose has a positive effect on body weight and walking ability, which is included in the sub-items of the MNA-SF [10,23].

In this study, 67% of malnourished patients with hip fracture admitted to the convalescent rehabilitation ward showed improved nutritional status during their hospital stay. This figure is lower than the 91% improvement in nutritional status documented in patients with stroke admitted to a convalescent rehabilitation ward [24]. A possible explanation for this difference may be that the patients in the present study were older. At present, the average age of patients with hip fracture admitted to convalescent rehabilitation wards in Japan has been reported to be 79.6 years and that of stroke patients to be 73.1 years [25]. Moreover, the average age of patients with hip fracture has been increasing year by year, and it will be necessary to increase the measures taken to prevent malnutrition in the future. The average length of hospital stay has been reported to be 54.9 days for patients with hip fracture and 83.3 days for patients with stroke [25]. Therefore, the effect of nutritional therapy during hospitalization may be more difficult to confirm in patients with hip fracture, who tend to have a shorter hospital stay. Furthermore, patients with hip fracture undergo highly invasive surgery, in addition to the invasive nature of the fracture itself, which may be associated with a hypermetabolic state during the period of nutritional management [26]. Under the health care insurance system in Japan, patients with hip fracture can stay in a convalescent rehabilitation ward for up to 90 days, which means that their nutrition status needs to be improved rapidly within the time window available. It has been reported that dietary intake in patients with hip fracture is increased more by multidisciplinary nutritional care than by conventional nutritional care [27]. In 2020, medical fees for a stay in a Japanese convalescent rehabilitation ward were revised to require that a dietitian with the highest possible certification is working full-time on the ward, and their greater collaboration with other rehabilitation therapists is acknowledged as a future requirement.

This study has several limitations. First, the rehabilitation dose was analyzed as a total quantity and did not examine the distribution of rehabilitation doses in terms of physical therapy, occupational therapy, and speech–language–hearing therapy. Second, the qualitative content of the rehabilitation could not be investigated and will need to be investigated in the future. Third, we have not collected and adjusted data on socioeconomic factors such as education, marriage status, number of family members, and household income that could affect nutritional status. Finally, daily protein intake is not included in the JRND; therefore, relevant survey results are not available. Given that protein intake may be directly related to improved nutritional status, further research in this area is needed.

## 5. Conclusions

In malnourished patients with hip fracture, admitted to a convalescent rehabilitation ward, increased daily rehabilitation dose improved nutritional status at discharge in 67% of cases. It is essential to ensure a balance between nutritional therapy and rehabilitation dose to improve nutritional status in these patients.

## Figures and Tables

**Table 1:** Patient characteristics overall and by study group at baseline.

Variables	Overall (*n* = 145)	Nutritional Status Improved (*n* = 97)	Nutritional Status Not Improved (*n* = 48)	*p*-Value
Age, years, mean ± SD	85.1 ± 7.9	84.2 ± 6.9	86.9 ± 9.6	0.009 ^a^
Female sex, *n* (%)	118 (81)	80 (82)	38 (79)	0.630 ^c^
CCI, median (IQR)	1 (0–2)	1 (0–2)	1 (1–2)	0.225 ^b^
Admission FIM score, median (IQR)	63 (46–81)	71 (55–85.5)	46 (33.3–64.5)	<0.001 ^b^
Admission MNA-SF score, median (IQR)	5 (4–6)	5 (4–6.5)	4 (3–5.8)	<0.001 ^b^
Admission FILS score, median (IQR)	9 (8–10)	9 (8–10)	8 (7–9.8)	<0.001 ^b^
Energy intake, kcal/day, median (IQR)	1400 (1160–1400)	1400 (1260–1490)	1200 (1008.5–1400)	<0.001 ^b^
Daily rehabilitation dose, min/day, median (IQR)	109.5 (94.6–116.2)	110.6 (103.3–117.9)	101.11 (77.4–113)	0.008 ^b^
Hospital stay, days median (IQR)	71 (50.5–85)	73 (50.5–85)	67.5 (50–84.8)	0.696 ^b^
Number of hospital beds, median (IQR)	104 (50.5–206)	206 (60–206)	60 (40–206)	0.012 ^b^

SD, standard deviation; CCI, Charlson Comorbidity Index; IQR, interquartile range; FIM, Functional Independence Measure; MNA-SF, Mini Nutritional Assessment-Short Form; FILS, Food Intake LEVEL Scale; ^a^ unpaired *t*-test; ^b^ Mann-Whitney U test; ^c^ chi-squared test.

**Table 2:** Spearman’s rank correlation coefficient for each survey item.

Variables	Age	CCI	Admission FIM	Admission MNA-SF^®^	Admission FILS	Energy Intake	Daily Rehabilitation Dose	Length of Hospital Stay	Number of Hospital Beds
Age	1	−0.079	−0.303 **	−0.159	−0.216 **	−0.210 *	0.028	0.295 **	0.061
CCI		1	−0.125	−0.058	−0.128	−0.020	0.018	0.050	−0.032
Admission FIM			1	0.359 **	0.514 **	0.330 **	0.173 *	−0.315 **	0.255 **
Admission MNA-SF^®^				1	0.413 **	0.270 **	0.062	−0.174 *	−0.120
Admission FILS					1	0.357 **	0.055	−0.160	0.074
Energy intake						1	0.151	−0.091	0.283 **
Daily rehabilitation dose							1	0.135	0.315 **
Length of hospital stay								1	0.045
Number of hospital beds									1

CCI, Charlson Comorbidity Index; FIM, Functional Independence Measure; MNA-SF, Mini Nutritional Assessment-Short Form; FILS, Food Intake LEVEL Scale; * *p* < 0.05; ** *p* < 0.01.

**Table 3:** Logistic regression analysis of the effect of daily rehabilitation dose on improved nutritional status at hospital discharge.

Variables	OR	95% CI		*p*-Value
		Lower	Upper	
Age	0.970	0.914	1.030	0.321
Sex	0.448	0.140	1.438	0.177
CCI	0.866	0.588	1.275	0.467
Admission FIM score	1.042	1.016	1.068	0.001
Admission MNA-SF score	1.349	0.991	1.837	0.057
Admission FILS score	0.987	0.684	1.423	0.944
Energy intake	1.002	1.000	1.004	0.048
Daily rehabilitation dose	1.023	1.002	1.045	0.031
Length of hospital stay	1.026	1.003	1.049	0.023
Center	1.234	0.976	1.562	0.079
Number of hospital beds	1.001	0.995	1.008	0.742

OR, odds ratio; CI, confidence interval; CCI, Charlson Comorbidity Index; FIM, Functional Independence Measure; MNA-SF, Mini Nutritional Assessment-Short Form; FILS, Food Intake LEVEL Scale.

## Data Availability

The data presented in this study are available on request from the corresponding author.
